# Synergistic induction of tertiary lymphoid structures by chemoimmunotherapy in bladder cancer

**DOI:** 10.1038/s41416-024-02598-7

**Published:** 2024-02-08

**Authors:** Lu Zhang, Ruiyun Zhang, Di Jin, Tianxiang Zhang, Akezhouli Shahatiaili, Jingyu Zang, Lu Wang, Yuanchun Pu, Guanglei Zhuang, Haige Chen, Jinhai Fan

**Affiliations:** 1 Department of Urology, The First Affiliated Hospital of Xi’an Jiaotong University, Xi’an, China; 2 Department of Urology, State Key Laboratory of Systems Medicine for Cancer, Ren Ji Hospital, Shanghai Jiao Tong University School of Medicine, Shanghai, China; 3 Department of Radiation Oncology, Ren Ji Hospital, Shanghai Jiao Tong University School of Medicine, Shanghai, China; 4 Shanghai Key Laboratory of Gynecologic Oncology, Ren Ji Hospital, Shanghai Jiao Tong University School of Medicine, Shanghai, China

**Keywords:** Cancer immunotherapy, Bladder cancer

## Abstract

**Background:**

A substantial number of patients with bladder cancer fail to benefit from immune checkpoint inhibitors (ICIs). We aim to investigate whether the addition of other therapeutic modalities into immunotherapy may augment the immune reactivity, thereby improving the overall response rate.

**Methods:**

We conducted a comprehensive assessment of the immunological changes following immunotherapy and chemotherapy, employing both single-cell RNA sequencing and bulk RNA sequencing analyses.

**Results:**

The bladder cancer patient treated with ICIs exhibited a higher abundance of B cells and T follicular helper cells compared to the treatment-naïve patient. Analysis of public datasets and the in-house RJBLC-I2N003 cohort revealed the induction of tertiary lymphoid structure (TLS) neogenesis and maturation by immunotherapy. The IMvigor 210 study suggested that TLS could serve as a predictor of immunotherapy response and patient prognosis. In addition, genome-wide transcriptome data unveiled a shift towards the immune-enriched subtype over the desert subtype in patients receiving neoadjuvant chemotherapy. Notably, the proportions of CD20 + B cells, T follicular helper cells, and TLSs were significantly increased. In patients treated with a combination of neoadjuvant chemotherapy and ICIs, TLS positivity and maturity were improved compared to the baseline. Furthermore, neoadjuvant chemoimmunotherapy resulted in a higher rate of pathological complete response compared to monotherapies.

**Conclusions:**

This work pinpointed the individual effect of immunotherapy and chemotherapy in fostering TLS development, and underscored the superior effectiveness of combined modalities in enhancing TLS maturation and response rates.

## Introduction

Bladder cancer ranks among the top ten malignancies, contributing to approximately 573,000 new cases and 213,000 deaths globally in 2020 [1]. Twenty-five percent of the patients are diagnosed with muscle-invasive bladder cancer (MIBC) [2]. For those with aggressive and resectable MIBC, neoadjuvant chemotherapy (NAC) followed by radical cystectomy is recommended by the 2020 guidelines of the European Association of Urology [3]. Recent years have witnessed remarkable advances in immunotherapy targeting immune checkpoint molecules, transforming the therapeutic landscape for bladder cancer. Cumulative clinical trials showcase unprecedented pathological responses and prolonged survival time in patients undergoing immunotherapy [4–7]. However, monotherapy often falls short of providing a lasting objective response [8], prompting extensive investigations of combination approaches to maximize the antitumor immunity for synergistic efficacy in MIBC patients.

Tertiary lymphoid structures (TLSs) contain an inner zone of CD20 + B cell follicle juxtaposed with a CD3 + T cell-rich zone [9, 10], and are commonly recognized as lymphocyte niches at the tumor site [11, 12]. Unlike canonical secondary lymphoid organs (SLOs), TLSs in the close vicinity of malignant lesions facilitate the trafficking and presentation of neighboring tumor antigens through dendritic cells (DCs) to educate T and B cells. This results in the efficient generation of antitumor immune reactions [13–15]. Therefore, it is tempting to speculate that therapeutic interventions triggering TLS neogenesis may enhance adaptive immune responses and boost cancer immunotherapies. Indeed, preclinical studies demonstrate that antiangiogenic therapies and immune checkpoint inhibitors (ICIs) stimulate intratumoral cytotoxic T-cell infiltration, leading to TLS formation and subsequent tumor regression [16, 17]. Additionally, TLS induction following tumor vaccination has been observed in regressing high-grade cervical intraepithelial neoplasia and pancreatic cancer lesions, supporting the idea that TLS neogenesis and tumor cell destruction depend on antigen-specific immune responses [12, 18, 19]. While previous studies have demonstrated the prognostic and predictive potentials of TLSs [20–24], it remains unclear whether conventional therapies, combined with immunotherapeutic agents, can converge to induce TLSs and enhance antitumor immunity in human cancers.

In this study, we leveraged single-cell RNA sequencing (scRNA-seq) analysis, bulk RNA sequencing (RNA-seq), and tissue microarray (TMA) to illustrate that TLS neogenesis could arise upon ICI monotherapy and NAC. Hematoxylin and Eosin (H&E) staining and multiplex immunofluorescence assays provided evidence that both immunotherapy and chemotherapy markedly improved TLS maturation. Combination therapy further increased the emergence of mature TLSs and the complete pathological response rate, supporting future extensive validation in larger cohorts.

## Materials and methods

### Patients and samples

All samples in this study were obtained from Ren Ji Hospital, and each patient provided written informed consent. The Ren Ji Hospital Ethics Committee approved the study. The study included three MIBC cohorts with neoadjuvant therapy: a cohort of 92 cases with neoadjuvant gemcitabine plus cisplatin chemotherapy (NAC cohort), a cohort previously reported of 20 cases with neoadjuvant toripalimab immunotherapy (RJBLC-I2N003, registered at http://www.chictr.org.cn, ChiCTR2000029500) [25], and a cohort of 41 cases with NAC and immunotherapy. Single-cell RNA sequencing was performed on two samples (one pre-ICI sample and one post-ICI sample), and bulk RNA sequencing was performed on 30 patients (30 pre-NAC samples and 22 post-NAC samples). Ninety-two patients (92 pre-NAC samples and 60 post-NAC samples) with formalin-fixed and paraffin-embedded specimens were subjected to tissue microarray. The Gene Expression Omnibus (GEO, http://www.ncbi.nlm.nih.gov/geo/) database was assessed to obtain the published GSE91061 [26], GSE115821 [27], and IMvigor 210 ^6^ datasets, and the IMvigor210CoreBiologies R package (v. 1.0.0) was utilized.

### Single-cell sample preparation and sequencing

Single-cell RNA sequencing was performed on tumor biopsy specimens obtained from two MIBC patients. The cell suspension was generated according to the 10x Genomics Single Cell Protocols. Subsequently, cells were barcoded with a Chromium Single-Cell Controller Instrument (10x Genomics). RNA from the barcoded cells was reverse-transcribed, and sequencing libraries were constructed with Chromium Single Cell 3’ Reagent v3 kits according to the manufacturer’s instructions. Sequencing was performed on an Illumina HiSeq 2000 system.

### Data processing and quality control

The Cell Ranger software was used to demultiplex cellular barcodes, map reads to the transcriptome, and down-sample reads as required to generate normalized aggregate data across samples. The process produced a raw unique molecular identifier (UMI) count matrix which was analyzed using the R package Seurat (version 4.0.2). Cells with over 20% mitochondrial-derived UMI counts were considered low-quality and removed. After quality control, the remaining cells were used in the downstream analyses.

### RNA sequencing

We performed RNA sequencing on 30 patients (30 pre-NAC samples and 22 post-NAC samples). Total RNA was extracted from tumor tissues with RNeasy plus kit (Qiagen) according to the manufacturer’s protocol. RNA purity and integrity were assessed by the NanoPhotometer spectrophotometer (Implant) and the RNA Nano 6000 Assay Kit of the Bioanalyzer 2100 system (Agilent Technologies). Three grams of each sample were subjected to RNA library preparation using the NEBNext Ultra Directional RNA Library Prep Kit (NEB). The index-coded libraries were clustered on a cBot Cluster Generation System using TreSeq PE Cluster Kit v3-cBot-HS (Illumina) and sequenced on an Illumina Hiseq X Ten platform to generate 125 bp paired-end reads (Novogene). Clean data were obtained from fastq raw data by removing adapter, ploy-N sequences, and low-quality reads. All the downstream analyses were based on clean data with high quality. The reference genome index was built using Bowtie v2.0.6, and paired-end clean reads were aligned to the reference genome (Ensembl hg38 human genome) using TopHat v2.0.9 [28]. The mapped reads were assembled using Cufflinks (v2.1.1) in a reference-based approach [29]. Differential expression analysis was conducted by Cufflinks (v2.1.1). Genes with a P-adjust value of 0.05 were considered differentially expressed genes.

### Molecular subtypes and signature scores

We classified the RNA-seq samples into consensus, TCGA, MDA, Lund, CIT, UNC, and Baylor subtypes using R packages consensusMIBC (v.1.1.0) available at https://github.com/cit-bioinfo/consensusMIBC and BLCAsubtyping (v. 2.1.1) available at https://github.com/cit-bioinfo/BLCAsubtyping [30]. We retrieved gene signatures in literature to identify tumor microenvironment-related subtypes [31]. The ConsensusClusterPlus R package was used for consensus clustering analysis [32]. We quantified the relative level of four TLS signatures by conducting the single-sample gene set enrichment analysis (ssGSEA) with the GSVA package [11, 33].

### Tissue microarray and immunohistochemistry

A tissue microarray (TMA) from 92 patients (92 pre-NAC samples and 60 post-NAC samples) was constructed. Tissue cylinders, with a diameter of 0.6 mm, were punched from morphologically representative areas at the tumor sites of each paraffin-embedded tissue block. A semi-automated tissue arrayer then placed these cylinders into a recipient paraffin block. Sections obtained from the TMA were stained with Hematoxylin and Eosin (H&E) to confirm the accurate position of the tumor lesion. Immunohistochemistry (IHC) was performed on these sections. Slides were baked, deparaffinized in xylene, passed through graded alcohols, and antigen retrieved with 10 mM citrate buffer, pH 6.0, in a steam pressure cooker. The slides were then treated with peroxidase block (Dako) to quench endogenous peroxidase activity, blocked using protein block (Dako), and incubated with primary antibodies. 50 mM Tris-HCl, pH 7.4, was used to wash the slides, followed by incubation with horseradish peroxidase-conjugated secondary antibodies. Immunoperoxidase staining was developed using the DAB system (Dako) according to the manufacturer’s instructions. Slides were counterstained with hematoxylin, dehydrated in graded alcohol and xylene, and cover-slipped using the mounting solution. The Aperio ScanScope system (Leica Biosystems) was used to scan and quantify the staining by Aperio ImageScope software v12.3.3.

### TLS quantification

TLSs were quantified based on H&E staining, and B-cell aggregates were quantified based on IHC (CD20 + B cell aggregates or islets) [10]. TLS positivity was defined as the TLS number ≥1. We used published criteria to distinguish the maturation stages of TLSs as follows: (1) early TLSs, ill-defined clusters of lymphocytes; (2) primary TLSs, round-shaped clusters of lymphocytes without a germinal center; (3) secondary TLSs, round-shaped clusters of lymphocytes with a germinal center [12, 24, 34]. In this study, immature TLSs referred to early TLSs and primary TLSs, while mature TLSs referred to secondary TLSs.

### Multiplex immunofluorescence assay

For multiplex immunofluorescence staining, we used the Opal staining method to probe the following markers: CD20, CD21, CD23, CD3, Ki67, and PanCK, with subsequent visualization using fluorescein 480, 620, 570, 520, 780, 690, respectively. Nuclei were visualized with DAPI. All slides were scanned using the ZEISS Axioscan7 Multispectral Imaging System and evaluated by ZEN 3.3 software.

### Statistical analysis

All statistical analyses were performed using R version 4.1.0. The ‘survival’ and ‘survminer’ packages were used to conduct Kaplan-Meier survival analysis. The deconvolution algorithms within the IOBR package (https://github.com/IOBR/IOBR) [35], including ESTIMATE, xCell, MCPcounter, and EPIC, were used to estimate the abundance of immune infiltration. CIBERSORTx [36] was used to quantify B cells infiltration in the NAC RNA-seq based on our scRNA-seq data. Two-sided Student’s *t* tests were used to compare continuous variables, while Fisher’s exact test and Chi-square test were applied to discrete variables. *P* values of < 0.05 were considered statistically significant.

## Results

### Identification of TLS induction upon immunotherapy via scRNA-seq analysis

**Fig. 1:**
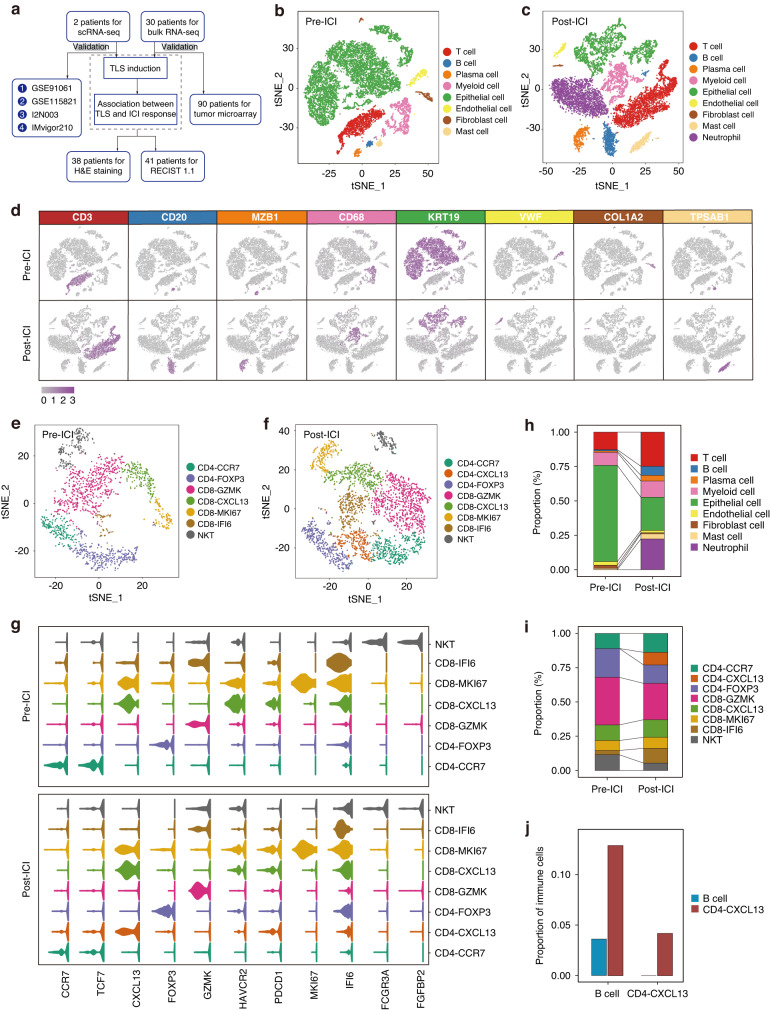
Identification of TLS induction upon immunotherapy via scRNA-seq analysis. **a** Flowchart of the overall study design. Cell type identification in pre-ICI (**b**) and post-ICI (**c**) samples, with tSNE plots depicting color-coded major cell types. **d** Feature plots illustrating marker genes of major cell types. T cell clusters in pre-ICI (**e**) and post-ICI (**f**) samples displayed by tSNE plots. **g** Violin plots representing marker genes of T cell clusters. **h** Relative abundance of major cell types in pre-ICI and post-ICI samples. **i** Relative abundance of T cell clusters in pre-ICI and post-ICI samples. **j** Comparison of proportions of B cells and CD4-CXCL13 T cells to total immune cells between pre-ICI and post-ICI samples.

To unravel the cellular changes following immunotherapy [37], we conducted scRNA-seq analysis on primary tumor tissues from two MIBC patients. One patient was treatment-naïve (pre-ICI) and the other received atezolizumab treatment (post-ICI) (Fig. 1a). A total of 13,989 cells from the pre-ICI sample were clustered into eight major cell types (Fig. 1b), and 13,951 cells from the post-ICI sample were clustered into nine major cell types (Fig. 1c), based on t-distributed stochastic neighbor embedding (t-SNE) dimensionality reduction. Dim plots (Fig. 1d) and violin plots (Supplementary Fig. 1A) depicted the expression levels of specific genes used for cell type annotation: T cell (CD3), B cell (CD20), plasma cell (MZB1), myeloid cell (CD68), epithelial cell (KRT19), endothelial cell (VWF), fibroblast (COL1A2), and mast cell (TPSAB1). T cell compartments were subsequently divided into seven subclusters in the pre-ICI sample (Fig. 1e; Supplementary Fig. 1B) and eight subclusters in the post-ICI sample (Fig. 1f; Supplementary Fig. 1C). In both samples (Fig. 1g), we detected naïve T cells (CD4-CCR7), regulatory T cells (CD4-FOXP3), effector T cells (CD8-GZMK), exhausted T cells (CD8-CXCL13), and proliferating T cells (CD8-MKI67). Notably, post-ICI sample exhibited a higher proportion of T follicular helper cells (CD4-CXCL13) than pre-ICI sample.

To evaluate the cellular changes in response to atezolizumab, we compared the two samples for cell proportion and observed a strong increase in T and B cells (Fig. 1h), as well as T follicular helper cells (CD4-CXCL13) (Fig. 1i), following immunotherapy. We identified that the ratios of B cells and T follicular helper cells to immune cells enhanced after immunotherapy (Fig. 1j). Given the reported relevance of B cells and T follicular helper cells as pertinent cofounders of TLS formation [9, 21], we hypothesized that increased TLS presence might be a result of immunotherapy intervention.

### Validation of TLS induction following immunotherapy

**Fig. 2:**
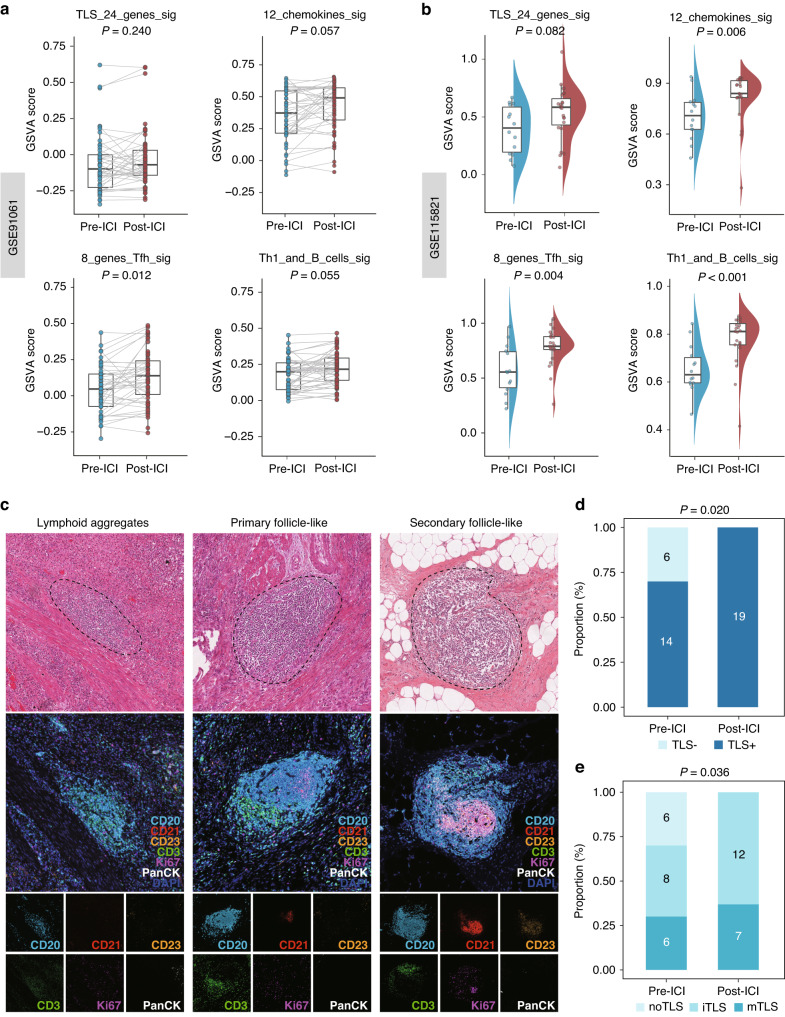
Validation of TLS induction following immunotherapy. **a** Comparison of GSVA scores utilizing four TLS signatures in 43 paired pre-ICI and post-ICI samples from GSE91061. **b** Comparison of GSVA scores utilizing four TLS signatures in pre-ICI and post-ICI samples from GSE115821. **c** Systematic evlauation of TLS maturity using H&E staining and multiplex IHC of CD20, CD21, CD23, CD3, Ki67 and PanCK. **d** Comparison of TLS positivity between pre-ICI and post-ICI samples in the RJBLC-I2N003 cohort. **e** Comparison of TLS maturity between pre-ICI and post-ICI samples in the RJBLC-I2N003 cohort.

To validate our hypothesis, we utilized two previously published immunotherapy datasets, GSE91061 and GSE115821, for gene set enrichment analysis (GSEA). Employing four established TLS gene signatures from the literature, including a 24-gene TLS signature, a 12-chemokine TLS signature, an 8-gene Tfh signature, and a Th1 and B cells signature [11], we quantified the TLS transcriptional abundance in these datasets. Our analysis consistently revealed an upregulation of these signatures following ICI treatment in both GSE91061 (Fig. 2a) and GSE115821 (Fig. 2b), affirming our scRNA-seq findings that immunotherapy might induce TLS formation. Additionally, our in-house RJBLC-I2N003 cohort yielded congruent results. When we systematically characterized TLS maturity through microscopic morphology in H&E staining and multiplex IHC staining (Fig. 2c) [12, 24, 34], the proportion of TLS positivity (Fig. 2d) and maturity (Fig. 2e) showed a marked increase after neoadjuvant toripalimab. These findings support our conclusion that immunotherapy could facilitate TLS neogenesis and maturation.

### Predictive value of TLS in immunotherapy

Accumulating evidence has indicated the predictive value of TLS in the context of ICIs [20, 21, 23]. Our results from both the GSE91061 dataset and the RJBLC-I2N003 cohort aligned with these observations. Specifically, in the GSE91061 dataset, we found that responders tended to exhibit higher TLS scores than non-responders at baseline (Supplementary Fig. 2A) and post-treatment (Supplementary Fig. 2B), although the trend did not reach statistical significance. We further categorized TLS signatures into high and low groups based on median scores, revealing a trend toward improved overall survival in patients with high scores at baseline (Supplementary Fig. 2C) and post-treatment (Supplementary Fig. 2D). The ROC curves also illustrated the promising value of TLS signatures in predicting immunotherapy response (Supplementary Fig. 2e). In the RJBLC-I2N003 cohort, we noted that MIBC patients with a higher incidence of TLSs, particularly mature TLSs, tended to have a favorable objective response as defined by RECIST 1.1 after immunotherapeutic treatment (Supplementary Fig. 3).

**Fig. 3:**
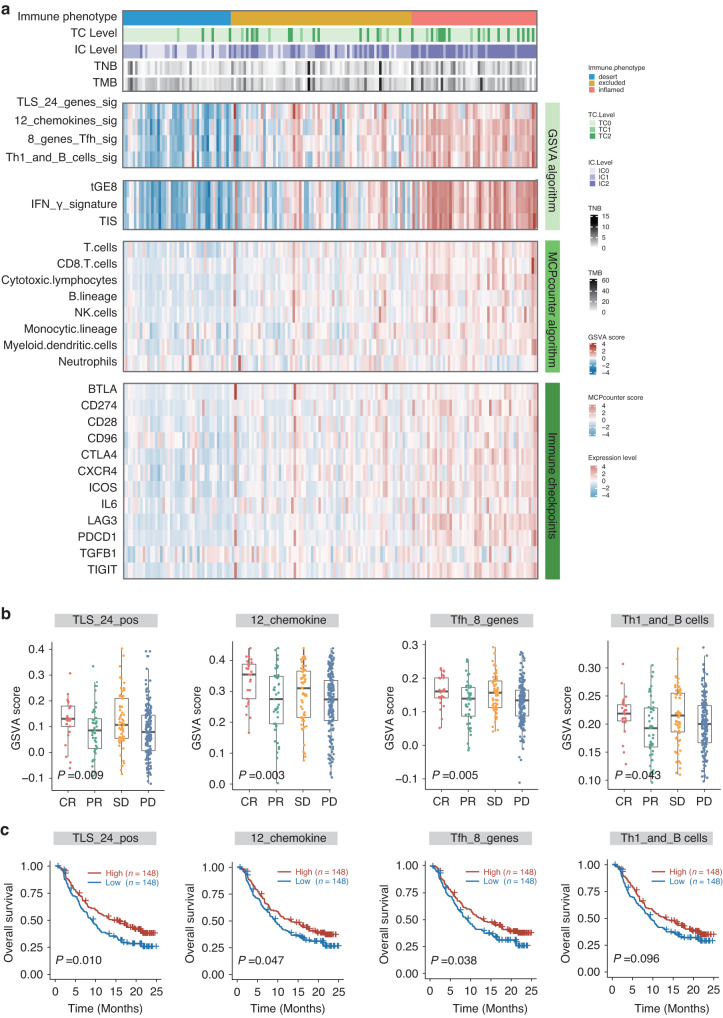
Predictive value of TLS in immunotherapy. **a** Heatmap illustrating the comprehensive landscape of immune phenotype, tumor cell (TC) level, immune cell (IC) level, tumor neoantigen burden (TNB), tumor mutation burden (TMB), TLS signature score, immune infiltrates signature score, immune cell population predicted by the MCPcounter algorithm, and immune checkpoint expression based on data from IMvigor210. **b** Box plots presenting GSVA scores of four TLS signatures stratified based on treatment responses. **c** Kaplan-Meier survival curves for ICI-treated patients from IMvigor210 stratified based on median GSVA scores of four TLS signatures.

To further illuminate the association between TLS and the immune response in MIBC, we leveraged RNA-seq data from the IMvigor210 clinical trial in urothelial carcinoma. Previous research has established positive correlations between tumor mutation burden, tumor-specific neoantigen, immune contexture, PD-L1 expression, and the response to immunotherapy. Our investigation revealed obvious correlations between TLS scores and these widely recognized proxy biomarkers for predicting immune response (Fig. 3a). Notably, complete responders showed the highest TLS scores, while patients with disease progression had the lowest TLS scores (Fig. 3b). Moreover, patients with high TLS scores experienced superior overall survival compared to those with low TLS scores (Fig. 3c). In summary, our findings suggest that TLS may serve as a promising surrogate for predicting the response and benefit to immunotherapy.

### Immune activation in patients undergoing neoadjuvant chemotherapy

**Fig. 4:**
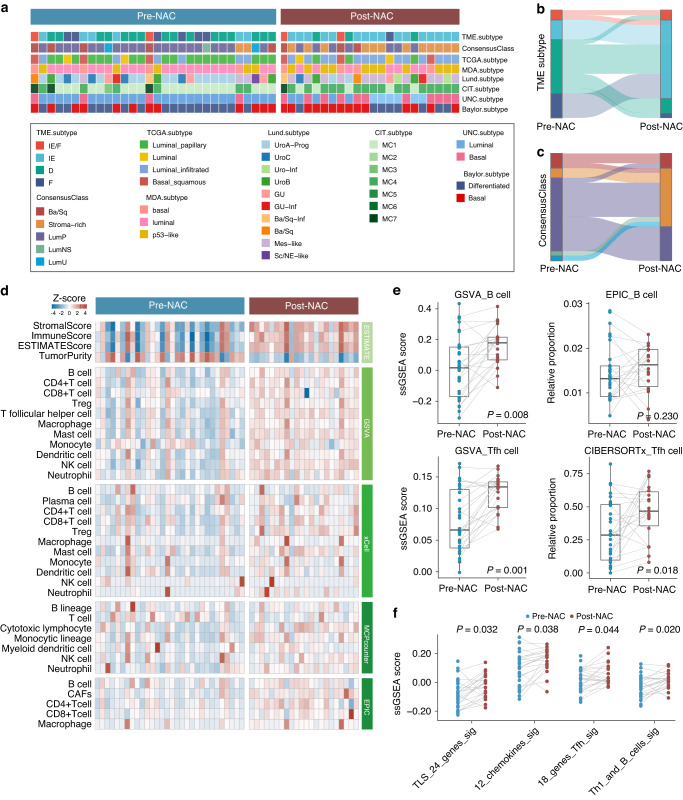
Immune activation in patients undergoing neoadjuvant chemotherapy. **a** Heatmap depicting the molecular subtypes assigned to each sample using multiple MIBC subtyping methods. **b** Sankey plot illustrating alterations in the TME subtype following NAC. **c** Sankey plot illustrating alterations in the MIBC consensusClass following NAC. **d** Heatmap displaying the differences in immune infiltrates between pre-NAC (*n* = 30) and post-NAC (*n* = 22) samples, as determined by various deconvolution algorithms. **e** Box plots presenting the relative levels of B cells and Tfh cells following NAC. **f** Comparison of GSVA scores utilizing four TLS signatures in paired pre-NAC and post-NAC samples.

The recognition of molecular subtypes in MIBC has proven pivotal for guiding therapeutic approaches and predicting responses to chemotherapy or immunotherapy [30, 31, 38, 39]. At the cellular and transcriptional levels, tumor microenvironment (TME) classification stands out as an effective method for deciphering the TME to predict immunotherapy responses [31]. To focus on the TME changes induced by NAC, we assessed molecular subtypes and TME classification by comparing pre-NAC samples with post-NAC samples (Fig. 4a). Our analysis revealed a shift towards immune-enriched subtypes over desert and fibrotic subtypes in TME classification (Fig. 4b), suggesting that NAC might elicit immune stimulation. Regarding consensus class stratification, the proportion of stromal-rich subtype increased after NAC treatment (Fig. 4c). A prior study has established that stromal-rich subtype harbors more TLS in MIBC [24]. Therefore, we reasoned that the immune contexture and subsequent TLS formation could be enhanced with chemotherapy, offering the potential to sensitize immunotherapy through inducing an immune-active TME when combined with chemotherapy intervention.

### Identification and validation of TLS induction following chemotherapy

To delve deeper into the TME alterations prompted by chemotherapy, we employed various transcriptional approaches to quantify the immune contexture in pre- and post- NAC samples. As anticipated, our analysis revealed a heightened abundance of immune cells in the TME landscape following chemotherapy, as indicated by multiple quantification algorithms (Fig. 4d). Notably, the increased presence of B cells and T follicular helper cells in patients undergoing NAC mirrored the observations in patients receiving immunotherapy (Fig. 4e). These findings led us to posit that chemotherapy might recruit B cells and T follicular helper cells to induce TLS formation. To bolster this hypothesis, we quantified TLS signatures in our NAC cohort and observed a significant increase in the post-NAC samples (Fig. 4f).

**Fig. 5:**
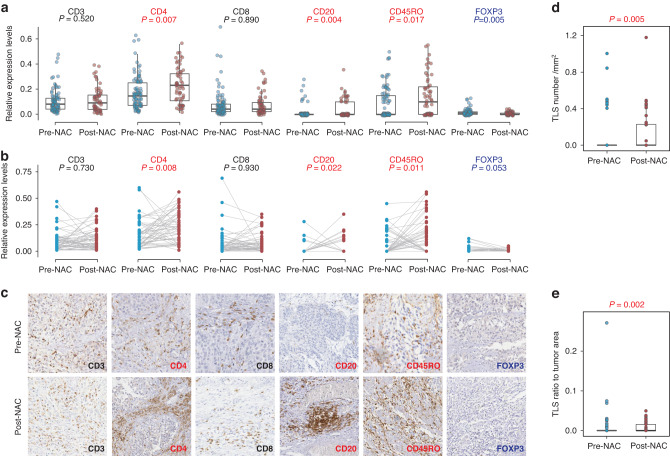
Identification and validation of TLS induction following chemotherapy. **a** Relative levels of IHC staining intensities in pre-NAC (*n* = 92) and post-NAC (*n* = 60) samples. Red labels indicate a significant decrease, blue labels indicate a significant decrease, and black labels indicate no significant changes. **b** Relative levels of IHC staining intensities in 54 paired pre-NAC and post-NAC samples. Red labels indicate a significant decrease, blue labels indicate a significant decrease, and black labels indicate no significant changes. **c** Representative IHC images of paired pre-NAC and post-NAC samples. **d** Comparison of TLS number relative to tumor area between pre-NAC and post-NAC samples. **e** Comparison of TLS size relative to tumor area between pre-NAC and post-NAC samples.

To corroborate these transcriptional findings at the protein level, we analyzed 152 samples in a tissue microarray by immunohistochemistry staining. In comparison to 90 baseline samples, the 62 post-NAC samples exhibited a significant increase in the density of CD4, CD20, and CD45RO, accompanied by a decrease in the density of FOXP3 (Fig. 5a). These findings were further confirmed through pairwise analysis of 54 paired samples (Fig. 5b) and were visually verified by representative IHC images (Fig. 5c). Furthermore, the post-NAC samples demonstrated a significantly higher number of TLS (Fig. 5d) and TLS density (Fig. 5e) compared to the pre-NAC samples. However, no association was found between NAC response and pre-NAC TLS (Supplementary Fig. 4A) or post-NAC TLS (Supplementary Fig. 4B), a conclusion supported by TLS quantification in pre-NAC (Supplementary Fig. 4C) and post-NAC (Supplementary Fig. 4D) TMA samples. In summary, our results affirm that chemotherapy has the potential to stimulate immune cells and shape an inflamed TME, providing compelling prospects for therapeutically inducing TLS in the setting of bladder cancer.

### Increased TLS maturation and treatment response with chemoimmunotherapy

**Fig. 6:**
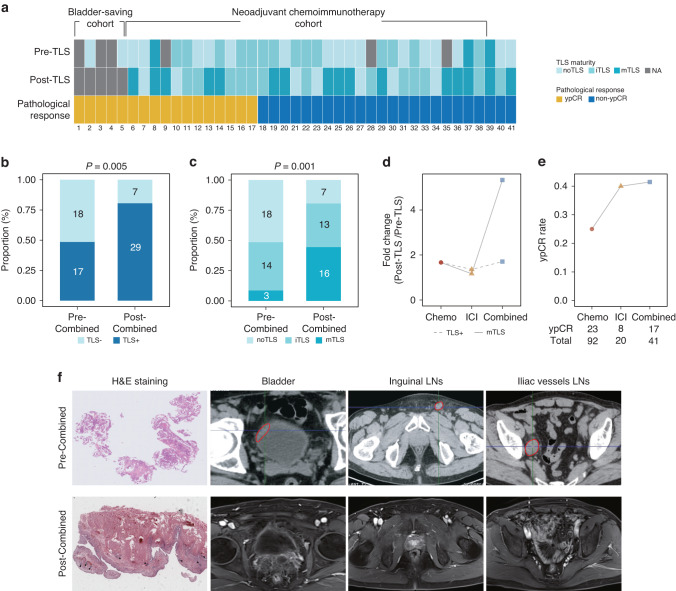
Increased TLS maturation and treatment response with chemoimmunotherapy. **a** Heatmap illustrating the characteristics of TLS maturation stages and pathological responses in patients receiving combined therapy. **b** Distribution of TLS positivity in baseline and post-treatment samples. **c** Distribution of TLS maturity in baseline and post-treatment samples. **d** Fold change of TLS positivity and maturity in tumors following chemotherapy, immunotherapy, and combined therapy. **e** Comparison of pathological complete response rates associated with neoadjuvant chemotherapy, immunotherapy, and combined therapy. **f** A representative case presenting a patient who developed mature TLSs after combination therapy and achieved a complete response.

In light of these insights, we speculated that the combined approach of chemotherapy and immunotherapy could yield synergistic therapeutic effects by inducing more TLSs. Initially, we examined TLS positivity and maturity in post-treatment samples in comparison to baseline samples (Fig. 6a). In line with our hypothesis, we found elevated proportions of TLS positivity (Fig. 6b) and maturity (Fig. 6c) in patients receiving combination therapy. More importantly, mature TLS induction was markedly enriched in the combination cohort compared to the monotherapy cohorts (Fig. 6d). Finally, we evaluated the objective response, defined by RECIST 1.1, across the three cohorts. Our study uncovered that combination treatment led to a superior pathological response compared to chemotherapy or immunotherapy alone (Fig. 6e). These results implied that the induction of mature TLSs might serve as a more effective surrogate for predicting immune response compared to TLS neogenesis. We noted that patients deriving mature TLSs from combination therapy could achieve a complete response in clinical practice (Fig. 6f), an observation warranting further investigation in larger cohorts.

## Discussion

In this study, we employed scRNA-seq analysis, bulk RNA-seq, and tumor microarrays to investigate the impact of ICIs and chemotherapeutic agents on TLS induction and maturation by taking advantage of unique MIBC cohorts. Our findings revealed that both treatment modalities could induce TLS neogenesis. Furthermore, the combined application of chemotherapy with immunotherapy demonstrated a synergistic effect, fostering enhanced TLS maturation and contributing to improved pathological responses. These results provide a compelling rationale to support the use of chemoimmunotherapy against bladder cancer.

Although atezolizumb and pembrolizumab have been approved by the U.S. Food and Drug Administration for bladder cancer since 2017 [40], only a limited subset of patients experience pronounced benefits from these immunotherapeutics, partially attributable to constrained preexisting immunoreactive landscapes [6, 7, 41–43]. The key question lies in if certain immunostimulatory interventions may elicit de novo or reactivate tumor-resident immune activites to augment immunotherapy efficacy. A mounting body of evidence suggests that TLSs possess the capacity to generate adaptative immune responses. Activated B cells within TLSs can release tumor-specific antibodies, triggering antibody-dependent cell death [44]. Additionally, preclinical investigations indicate that TLSs can reinvigorate T cell cytotoxic function [45, 46]. Furthermore, there is circumstantial evidence supporting the occurrence of adaptive immune reactions in TLSs in the absence of SLOs in murine models [47], unveiling functional similarities in enhancing immune reactivity between TLSs and SLOs. The substantial advantages associated with TLSs have prompted ongoing research endeavors aimed at therapeutically inducing TLSs to optimize immunotherapy responses, notably through approaches such as anti-angiogenic therapies [16, 17] and tumor vaccinations [18, 19]. In line with a recent report [21], we observed the induction of TLS neogenesis and maturation by immunotherapy. While mature TLSs at baseline have been identified as predictors of ICI efficacy [34], our findings of the emergence of mature TLSs upon immunotherapy in patients with favorable pathological responses complement and extend these prior literature. It would be interesting to determine the predictive value of post-treatment TLSs in a prospective setting.

NAC is the standard care for patients with resectable and non-metastatic MIBC [3]. While chemotherapy is known to exert direct cytotoxic effects on tumor cells, cumulative evidence suggests its ability to initiate antitumor immune responses [48]. Numerous studies demonstrate the immunostimulatory activities of chemotherapy, characterized by increased abundance of effector T cells and decreased frequency of regulatory T cells across various human tumors [49, 50]. In concordance with these observations, our analysis revealed a shift in the TME from immune-inert toward immune-enriched subtypes in the NAC cohort. Of note, we used computational deconvolution of bulk gene expression profiles and immunohistochemical assessment of TMA to substantiate the notion that chemotherapy could increase T and B cell infiltration, decrease regulatory T cells, and induce TLS neogenesis, which has only been sporadically reported in the past [51]. It is worthy to mention that we failed to identify a predictive role of TLS for chemotherapy outcome, in contrast to its well-established relevance in the context of immunotherapy. Considering that chemotherapy also directly targets tumor cells, this result might not be entirely surprising. However, we reasoned that the combination of chemotherapy and immunotherapy could synergistically facilitate TLS development. Consistent with our hypothesis, the addition of chemotherapy to immunotherapy instigated TLS initiation and maturation, thereby eliciting robust antitumor immunity and boosting pathological complete responses. Remarkably, patients manifesting mature TLSs were more prone to achieve complete responses, underscoring the imperative for clinical trials to evaluate the efficacy and biomarkers of combination strategies. Collectively, our research introduces a fresh perspective, positioning chemotherapy not only as a cytotoxic agent but also as an immunostimulatory modality, and advocates for the preference of combination therapy over monotherapy based on the theoretical underpinning of TLS activation. Such an important implication may be applicable to antibody-drug conjugates as an emerging class of bladder cancer treatment.

Several limitations of this study should be acknowledged. Firstly, despite the inclusion of multiple cohorts with a relatively large number of patients, there exists the potential for sampling bias inherent in the retrospective design. Secondly, the NAC cohort exclusively received gemcitabine plus cisplatin, the most commonly employed chemotherapy regimen for MIBC. Consequently, TLS induction by alternative chemotherapeutics requires further exploration. Thirdly, sequential immunotherapy upon chemotherapy induction, as opposed to concurrent chemoimmuntherapy, presents another avenue to enhance immunotherapy efficacy which warrants future investigations. Nevertheless, our study offers pioneering insights into biological mechanisms underlying clinical benefits arising from the integration of chemotherapy and immunotherapy, specifically in promoting TLS neogenesis and maturation.

### Supplementary information

Supplementary material

## Data Availability

Original data are available upon reasonable request.
