# Supplementary material for: Synergistic induction of tertiary lymphoid structures by chemoimmunotherapy in bladder cancer
Source: Br J Cancer. 2024 Feb 8;130(7):1221–31. doi: 10.1038/s41416-024-02598-7 (PMC10991273; doi:10.1038/s41416-024-02598-7)
Supplement: Supplementary file 1 — Supplementary material [file 41416_2024_2598_MOESM1_ESM.docx]

**Supplementary Material**

**
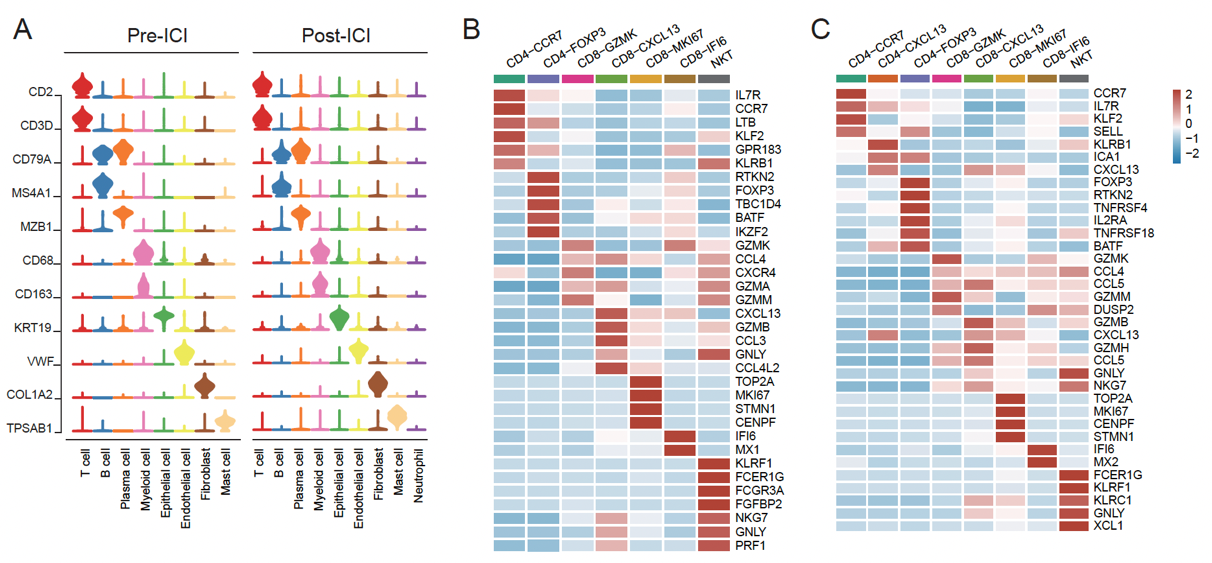
**

**Supplementary Figure 1: Identifying cell infiltrates in pre-ICI and post-ICI samples.**

**A** Violin plots for makers of major cell types. **B** Heatmap for representative markers of T clusters in the pre-ICI sample. **C** Heatmap for representative markers of T clusters in the post-ICI sample.


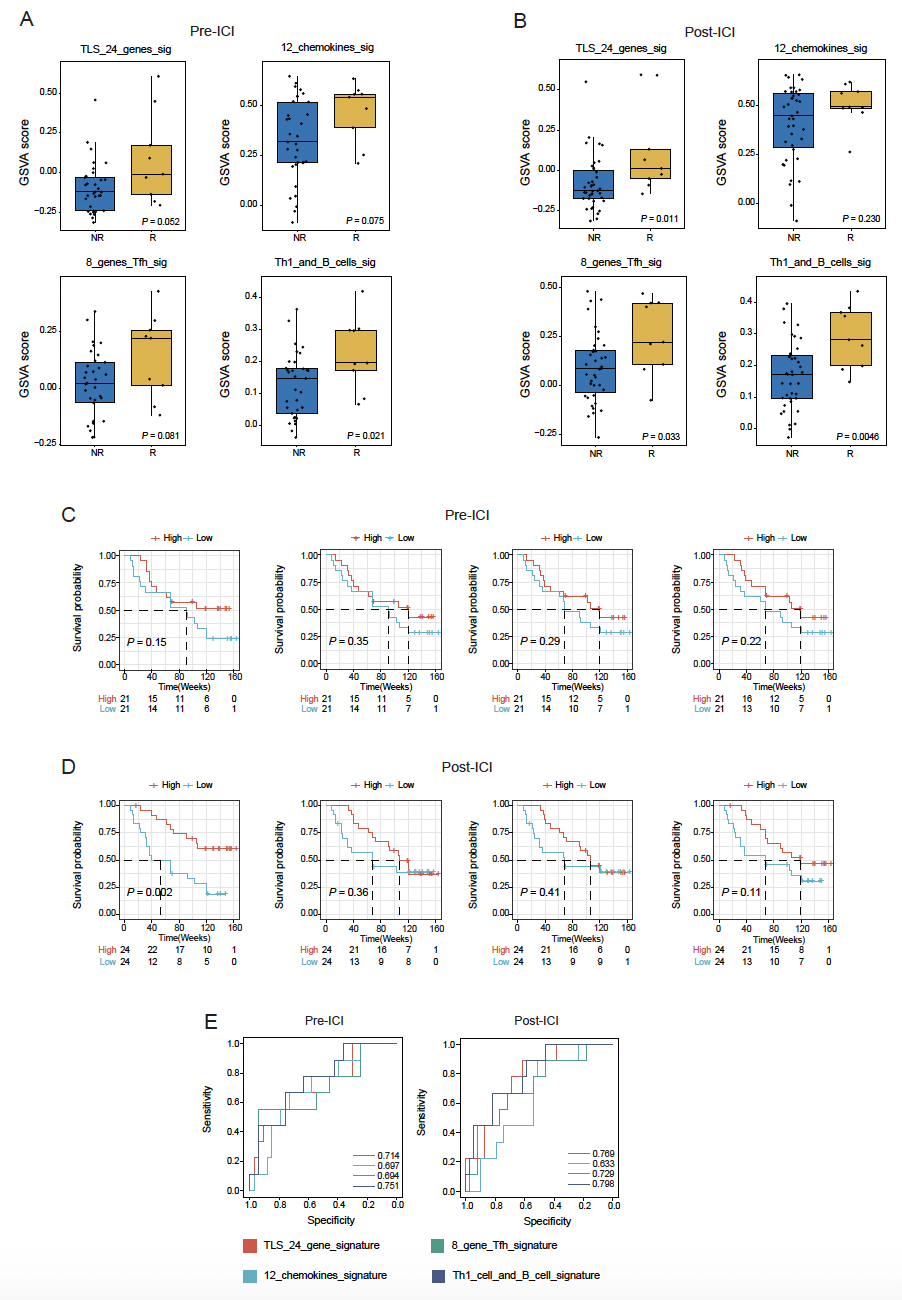


**Supplementary Figure 2: Association of TLS with immunotherapy response and survival time in GSE91061.**

**A** Boxplots showed the GSVA quantification of responders and non-responders stratified by TLS scores in pre-ICI samples (n=51). **B** Boxplots showed the GSVA quantification of responders and non-responders stratified by TLS signature scores in post-ICI samples (n=53). **C** Kaplan-Meier survival curves were stratified by 4 TLS signatures in pre-ICI samples. **D** Kaplan-Meier survival curves were stratified by 4 TLS signatures in post-ICI samples. **E** ROC curves of 4 TLS signatures were generated to predict the immunotherapy response in pre-ICI and post-ICI samples.

**
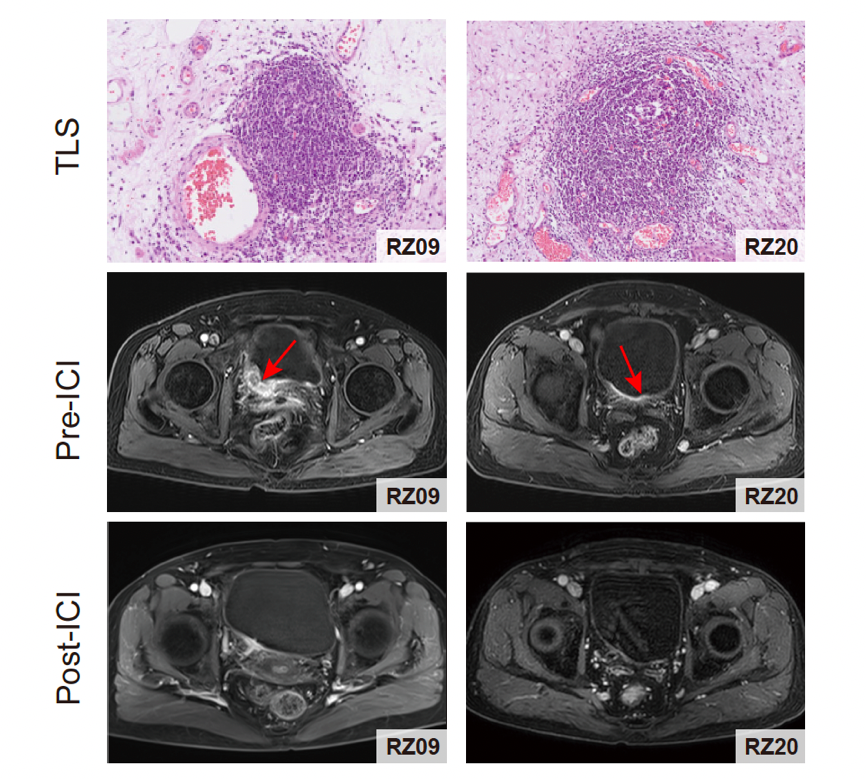
**

**Supplementary Figure 3: Mature TLS indicate favorable objective response**

Representative H&E staining and MRI images of RZ09 and RZ20 were presented for pathological and radiographic evaluations, respectively.


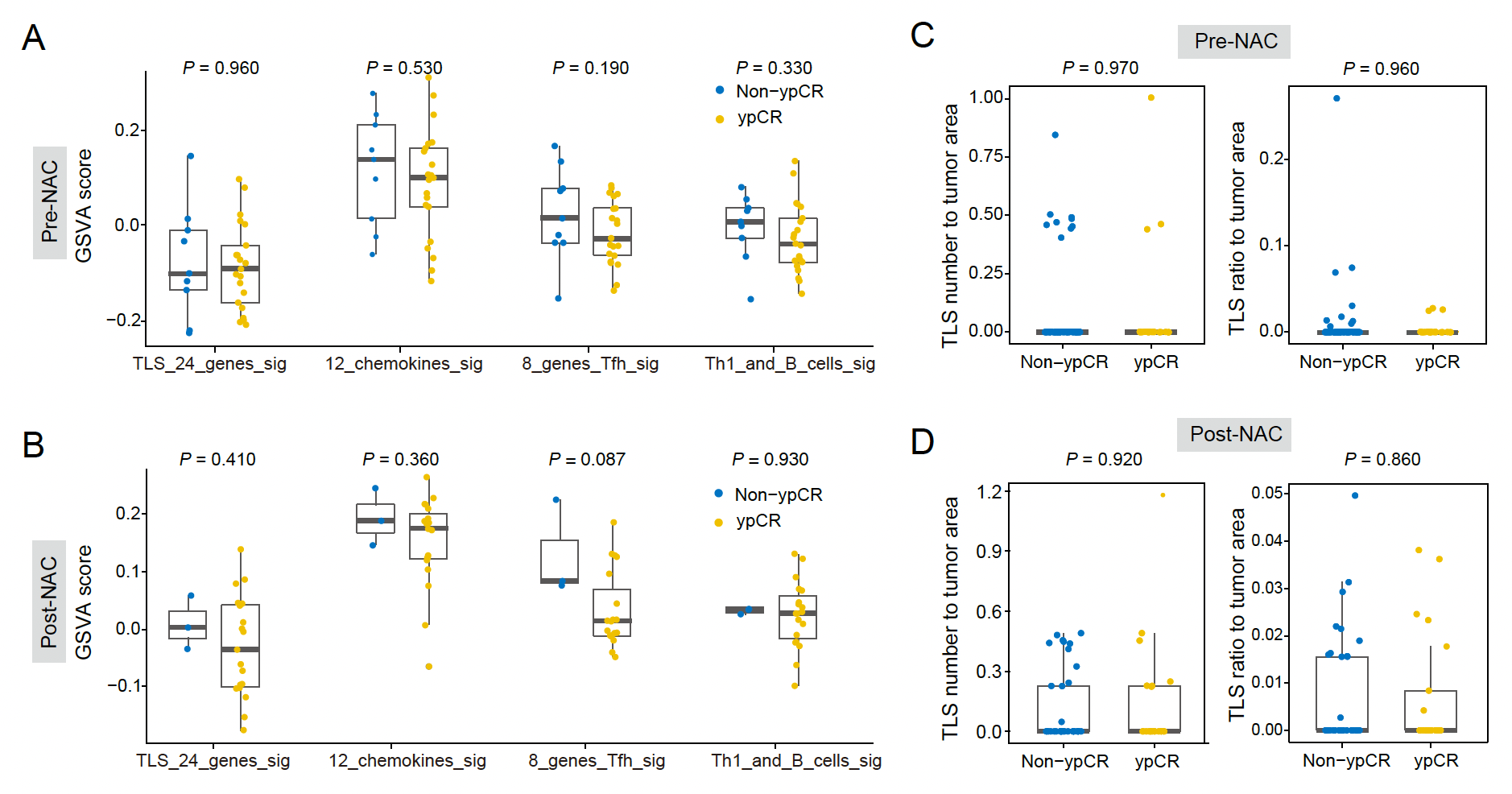


**Supplementary Figure 4:** **Association between TLS and NAC response**

**A** Bar plots showed TLS number and TLS density to tumor area in patients stratified by pathological responses before receiving NAC. **B** Bar plots showed TLS number and TLS density in patients stratified by pathological responses after receiving NAC. **C** Comparison of TLS scores between ypCR and non-ypCR patients at baseline. **D** Comparison of TLS scores between ypCR and non-ypCR patients after receiving NAC.
